# Measuring Nature Contact: A Narrative Review

**DOI:** 10.3390/ijerph18084092

**Published:** 2021-04-13

**Authors:** Isabel Holland, Nicole V. DeVille, Matthew H. E. M. Browning, Ryan M. Buehler, Jaime E. Hart, J. Aaron Hipp, Richard Mitchell, Donald A. Rakow, Jessica E. Schiff, Mathew P. White, Jie Yin, Peter James

**Affiliations:** 1 Channing Division of Network Medicine, Department of Medicine, Brigham and Women’s Hospital, Harvard Medical School, Boston, MA 02215, USA; rejch@channing.harvard.edu; 2 Department of Epidemiology, Harvard T.H. Chan School of Public Health, Boston, MA 02115, USA; 3 Department of Park, Recreation, and Tourism Management, Clemson University, Clemson, SC 29634, USA; mhb2@clemson.edu; 4 Division of Cancer Genetics and Prevention, Dana-Farber Cancer Institute, Boston, MA 02215, USA; RyanM_Buehler@dfci.harvard.edu; 5 Department of Environmental Health, Harvard T.H. Chan School of Public Health, Boston, MA 02215, USA; jschiff@hsph.harvard.edu (J.E.S.); jieyin@hsph.harvard.edu (J.Y.); pjames@hsph.harvard.edu (P.J.); 6 Department of Parks, Recreation, and Tourism Management, North Carolina State University, Raleigh, NC 27695, USA; jahipp@ncsu.edu; 7 Center for Geospatial Analytics, North Carolina State University, Raleigh, NC 27695, USA; 8 MRC/CSO Social and Public Health Sciences Unit, University of Glasgow, Glasgow G3 7HR, UK; Richard.Mitchell@glasgow.ac.uk; 9 Section of Horticulture, School of Integrative Plant Science, Cornell University, Ithaca, NY 14853, USA; dr14@cornell.edu; 10 European Centre for Environment and Human Health, University of Exeter, Cornwall TR1 3HD, UK; Mathew.White@exeter.ac.uk; 11 Urban and Environmental Psychology Group, University of Vienna, Vienna 1010, Austria; 12 College of Architecture and Urban Planning, Tongji University, Shanghai 200092, China; 13 Department of Population Medicine, Harvard Medical School and Harvard Pilgrim Health Care Institute, Boston, MA 02115, USA

**Keywords:** green space, greenness, nature, natural environment, nature exposure, exposure assessment, built environment, health, nature contact, nature dose

## Abstract

While many studies suggest evidence for the health benefits of nature, there is currently no standardized method to measure time spent in nature or nature contact, nor agreement on how best to define nature contact in research. The purpose of this review is to summarize how nature contact has been measured in recent health research and provide insight into current metrics of exposure to nature at individual and population scales. The most common methods include surrounding greenness, questionnaires, and global positioning systems (GPS) tracking. Several national-level surveys exist, though these are limited by their cross-sectional design, often measuring only a single component of time spent in nature, and poor links to measures of health. In future research, exposure assessment combining the quantifying (e.g., time spent in nature and frequency of visits to nature) and qualifying (e.g., greenness by the normalized difference of vegetation index (NDVI) and ratings on perception by individuals) aspects of current methods and leveraging innovative methods (e.g., experience sampling methods, ecological momentary assessment) will provide a more comprehensive understanding of the health effects of nature exposure and inform health policy and urban planning.

## 1. Introduction—Defining and Quantifying Time Spent in Nature

A growing body of epidemiological evidence suggests that exposure to nature is associated with improved health and well-being, including improved physical (e.g., lower mortality from cardiovascular disease) and mental health (e.g., reduced stress) [1,2,3,4]. However, given the sheer variety of nature experiences, there are currently no standardized methods or metrics used for measuring time spent in nature or nature contact, nor agreement on how best to define nature contact in research. Though nature is a complex construct, researchers broadly categorize nature exposure as any contact with vegetation or nonhuman animals in settings ranging from personal gardens to larger urban parks to relatively pristine wilderness [5,6]. Considering the many ways nature can be classified (e.g., forests, deserts, city parks, national parks, potted plants, back yards, bodies of water), defining and quantifying time spent in nature is a considerable challenge.

Nature contact varies largely at the individual and population level. Exposure assessment often utilizes simple metrics of availability (e.g., quantity of vegetation as measured by satellite imagery), accessibility (e.g., distance to green space), or visibility (e.g., amount of green space physically visible from a particular location such as the home), among others [4,7]. More nuanced measures of exposure (e.g., measures that incorporate multiple factors including the frequency, duration, and intensity of exposure and biological complexity) may better capture individuals’ lived experiences of the natural world. Such factors are integral components of nature exposure that ultimately impact health. The amount of green space in one’s neighborhood, the distance from one’s residence to the nearest green space or park, or the amount of time spent in natural environments all offer a measurable insight into an exposure–response relationship of nature and health. However, it is critical to consider the potential differential benefits to health depending on whether metrics capture only or more than one of these components of nature exposure.

Furthermore, potential pathways by which nature exposure may influence health include lower noise, reduced air pollution, and cooler temperatures, and those who do not actually spend time in but may live near to a park could still benefit. Alternative mechanistic pathways, such as physical activity, social connectivity/cohesion, or the microbiome, depend on an individual being physically present in the green space. The framework to quantify the link between exposure to nature and health outcomes across multiple pathways holds important implications for the design of health interventions (e.g., park prescriptions or nature exposure guidelines) and urban planning (e.g., prioritizing the most impactful natural features for physical activity). Population-level data on time spent outdoors (a commonly used metric collected by self-report) is one indicator of global nature exposure and a pathway that can be further investigated to establish an association between time in nature and health on a large scale.

In this narrative review, we assess the literature on measuring time spent in nature, describe current population-level estimates of time spent in nature, and discuss the strengths, limitations, and future research directions in this area. It should be noted that time spent in nature may not be the only important metric when considering pathways between nature exposure and health; however, examining other metrics is outside the scope of this review.

## 2. Methods

Narrative reviews are a type of qualitative research synthesis describing the results of quantitative studies that have used diverse methodologies or theoretical conceptualizations without a focus on the statistical significance of the findings [8,9]. We conducted a keyword search-based literature review using PubMed Advanced Search on 31 August 2020, for studies with abstracts or titles containing terms such as “nature”, “nature dose”, “greenness”, “green space”, “greenspace”, “outdoors”, “outdoor time/playtime” and “time-activity pattern”. We limited this review to research on human subjects only and included English language-based international peer-reviewed articles (e.g., primary research, reviews, commentaries), online reports, electronic books, and press releases. We also applied a snowballing search methodology using the references cited in the articles identified in the literature search. Based on the available studies at the beginning of this search, we focused our review on studies performed in the United States, Canada, the United Kingdom, Australia, and New Zealand. Each identified item was assessed for relevance by a member of the study team. This review is intended to summarize recent literature on quantifying exposure to nature and present current population-based metrics and is not meant to be exhaustive.

## 3. Results

In retrieving literature on methods of measuring time spent in nature, we reviewed a range of research from multiple disciplines, geographic regions, and study populations. The results presented below represent more recent literature (e.g., the last decade) primarily from Western countries. Furthermore, the focus of population-level estimates of time spent in nature was on the following countries: United States, Canada, United Kingdom, Australia and New Zealand. Though other countries may conduct regular or occasional population-level surveys of time spent in nature, those were outside the scope of this review.

### 3.1. Methods of Measuring Time Spent in Nature

#### 3.1.1. Surrounding Greenness and Green Space Access

Surrounding greenness and access to green space are often used as proxies for time spent in nature. These metrics provide information on the intensity of exposure as opposed to frequency and duration. Surrounding greenness, a quantification of the proportion of vegetation within a spatial unit (e.g., a defined area around a participant’s residential address), is widely used to approximate exposure to nature [10,11]. The most commonly utilized method for calculating greenness is the normal difference vegetation index (NDVI). NDVI computes the ratio of the difference between the near-infrared region (strongly reflected by vegetation) and red light (absorbed by chlorophyll in plants). NDVI is a standardized method to measure healthy vegetation, with larger values indicating higher levels of vegetative density [12], and estimates the generalized availability of vegetation in a particular area (e.g., residence, work, school), which may influence the likelihood that individuals spend time in or view natural environments. Furthermore, the methods to measure greenness (e.g., NDVI) are standardized and can be applied on local, regional, and global scales. Thus, exposure can be compared at the neighborhood, city, or country level. However, there are limitations of using NDVI as a proxy for time spent in nature. Time spent in nature may have different health effects if there is ambient greenery throughout the study area versus a higher concentration of greenery in one specific location (e.g., one local park), and NDVI values do not capture such differences. Thus, surrounding NDVI fails to provide information on the type of, quality of, access (e.g., physical, visual) to, and experience with available vegetation, does not incorporate time-activity patterns, and yields no insight into whether individuals are actually spending time in natural environments.

Another common proxy used by researchers to measure time spent in nature is green space access. Green space is an umbrella term describing land that is partially or totally covered with grass, trees, shrubs or other vegetation, ranging from home gardens to city parks to national parks [13]. Green spaces also include vacant lots overgrown with weeds, which could influence health differently than a well-maintained park, depending on the health pathway of interest [14]. A recent review of green space research across multiple disciplines highlights two common interpretations of green space: green space as nature or green space as urban vegetated space [15]. Of the studies that provided explicit definitions, Taylor and Hochuli identified six types that demonstrate the variability in how researchers conceptualize green space: a range of what is considered green space, definition by examples, ecosystem services, “green” or “natural” areas, generic land uses, and vegetated areas [15]. Green space accessibility measures have been used to estimate exposure to nature as a function of distance from the nearest green space, whether it be an urban public park or a forest [16]. However, green space access measures often do not account for amenities in a green space, and there is little agreement about which areas can be classified as green space. For instance, is agricultural land considered green space? Furthermore, current metrics of greenness and green space proximity do not typically take into account physical or other barriers (e.g., private property/facilities) that prevent accessibility to the study area of interest. For example, the Euclidean (i.e., straight-line) distance between a residence and a park may be very small; however, there could be a barrier such as a train track or fencing that physically prevents an individual from accessing the nearby green space [17].

The assumption is that these metrics will be correlated with the amount of time individuals spend in nature, where closer proximity to green space is associated with an increased probability of spending time in nature [18]. However, this assumption may be incorrect. Several studies illustrate no or weak associations between the availability of and proximity to green space and the use of the green space for physical activity [19,20,21]. These findings suggest another component of nature exposure (e.g., the quality or aesthetics of green space) may be influencing whether individuals spend time in nearby nature.

#### 3.1.2. Surveys of Time Spent in Nature

While metrics such as NDVI and green space access provide an estimate for the generalized availability of nature within an area, these measures do not capture details of personalized exposure such as timing, duration, frequency, seasonality and intensity. Shanahan et al. [22] proposed a nature-dose framework to quantify the link between health outcomes and experiences in nature, as measured by intensity (i.e., the quality and quantity of nature), frequency, and duration. Variation across these three factors may have an influence on the relationship between nature contact and health and are therefore important to quantify. Self-reporting via questionnaire is a frequently utilized methodology to capture any or all of these components at an individual level.

When measuring “nature exposure,” researchers typically describe the “intensity of greenness” (i.e., through metrics such as NDVI), while ignoring other essential aspects of exposure assessment, such as duration and frequency. Two important studies captured all three areas of nature-dose using questionnaires as their primary methodology. Shanahan et al. [22] considered the frequency, duration, and intensity of nature contact when examining health outcomes of depression, high blood pressure, social cohesion, and physical activity. Respondents were invited to report on any visit within the previous week to a place they considered “outdoor green space” and were asked to name or describe the location. The duration (i.e., average time per visit across the survey week) and frequency (i.e., green space visits across a year) of experience were asked of 1538 residents of Brisbane, Australia, via an online questionnaire. Intensity was measured as vegetation complexity derived from light detection and ranging (LiDAR) maps of vegetation cover, where higher levels of vegetation complexity were achieved in green spaces with higher vegetation cover and more complex vegetation structure. The authors found that participants with longer visits to green spaces had lower rates of depression and high blood pressure, and those who visited more frequently reported greater social cohesion. The duration and frequency of green space visits were associated with higher physical activity. In this particular study, intensity (i.e., vegetation complexity) was not associated with any of the health outcomes; however, other studies have demonstrated positive relationships between perceived species richness and feelings of restoration [23,24].

Cox et al. [25] generated three similar measures of nature-dose from questionnaires distributed to 1023 residents of Southern England, UK: frequency and duration (i.e., time spent in their garden and public green spaces) and intensity (i.e., quantity of neighborhood vegetation cover). Respondents also self-reported information on mental health, physical health, social cohesion, and positive physical behavior. Dose-response analyses showed that minimum thresholds of weekly nature dose (i.e., five or more hours) were associated with substantially lower levels of depression. In a separate study examining the frequency, dose, and intensity of nature exposure and urbanicity, Cox et al. [26] found positive relationships between the frequency and duration of nature exposure and four health outcomes (i.e., depression, self-rated physical health, perceived social cohesion, physical activity), whereas the intensity of nature exposure was associated only with perceived social cohesion. These studies emphasize the need for different metrics of nature exposure to provide a more comprehensive picture of the relationship between nature exposure and health outcomes.

With the exception of Shanahan et al. [22] and Cox et al. [25], most research relying on participant self-reporting to quantify exposure to nature captured only one or two metrics of duration, frequency, and intensity, not all three. For example, White et al. [27] derived recreational nature contact, or time spent in natural environments, as a single measure of weekly duration in hours and minutes self-reported by participants in the Monitor of Engagement with the Natural Environment (MENE) Survey in the UK. They found that, compared to no nature contact, the likelihood of reporting good health or high well-being became significantly greater with contact ≥120 min a week, and that there was no additional benefit on reported health beyond 300 min per week. Leveraging data from a nationally representative US study, the National Health and Nutrition Examination Survey (NHANES), researchers examined associations between time spent outdoors measured during a typical weekday and weekend and mental health and chronic disease outcomes [28,29]. This study found that time spent outdoors, on both weekdays and weekends, was associated with mental health benefits (e.g., fewer depressive symptoms) [28], less time spent sedentary, more time spent in moderate-to-vigorous physical activity, and a lower risk of chronic disease [29]. A recent review of studies in which participants spent time actively (e.g., walking) and passively (e.g., sitting or resting) in different natural environments highlights that varying nature dose (i.e., time spent outdoors ranging from 15 to 50+ min) yields physiological benefits and improved measures of affect and attention [30]. In other large-scale national surveys, participants were most commonly asked to report hours in a typical week spent outdoors (i.e., in nature), hours in the week preceding spent outdoors, or average hours per week spent doing outdoor recreation activities [31,32,33]. Other intervention studies relied on 24-h recall diaries to report time-activity patterns [34,35].

Studies, which are most often longitudinal, have commonly investigated outdoor play or time spent outdoors, as opposed to time in nature, in cohorts of children [36,37,38,39,40]. Parents or teachers are often asked to report how much time the child was playing outdoors in a typical day/week/month or how often children engaged in outdoor play in the past month. For instance, a U.S.-based study examined the effects of 49 common after-school and weekend programs in green outdoors, built outdoors, and indoor environments on children’s attention-deficit/hyperactivity disorder (ADHD) symptoms and found that green outdoor settings appeared to reduce ADHD symptoms across individual characteristics and geographic regions [41].

Self-report, particularly via in-person, telephone, or online surveys, allows for large sample sizes across broader, diverse populations. Questionnaires can provide information regarding the duration and frequency of time in nature and can also gather perceptions of quality of green space, safety, and self-reported engagement. In terms of limitations, questionnaires may introduce recall and/or social desirability bias. The question order can affect responses, and many factors have the potential to influence how well a person responds. Self-reported duration may also be less accurate than more objective measures obtained from geo-tracking individuals at single or multiple visits. It is important to note that while a selection of studies do explicitly ask about time spent in nature [22,25,31,42,43], time spent outdoors was more frequently reported, which does not necessarily measure time in a natural environment. Finally, even questionnaires that specifically ask about time spent in nature may not be comparable across different countries or populations, as cultural definitions and perceptions may impact how participants define nature.

#### 3.1.3. Global Positioning Systems (GPS) Tracking

GPS devices or GPS-enabled smartphones have emerged as novel methods in capturing exposure to nature by tracking an individual’s geographic location at a high temporal (e.g., once per second) and spatial (e.g., with 7 to 13 m accuracy) resolution [44]. Researchers can obtain specific data on where people went and the timepoints at each location through GPS tracking [45]. These coordinates can then be overlaid on NDVI or land use spatial datasets to derive metrics of time spent in nature for each participant.

One study, conducted as part of the Personal and Environmental Associations with Children‘s Health (PEACH) Project, examined green space and children’s physical activity for 1307 children in Bristol, UK for four days per participant [46]. The group found that 13% of monitored time was spent outdoors, but only 2% was spent in defined green space. During this outdoor time, 30% of activity volume and 35% of moderate-to-vigorous physical activity was accumulated. Studies such as this offer substantial high-accuracy data that provide valuable insight into where people spend their time, and when coupled with accelerometry data, may describe their behaviors in these locations [47,48].

GPS tracking, however, has its limitations. Studies using GPS devices can be hard to implement in large populations and may be subject to bias based on who is willing and able to be tracked. Furthermore, bias may be introduced when individuals know that their movements are being tracked and behave differently than they would when not undergoing observation. GPS locational accuracy, particularly in urban areas, can be poor; thus, devices (e.g., smart phones) may not be able to capture whether an individual is walking down the street outside of a park versus walking within the park. Due to the cost of the physical hardware used for tracking and the requirement for technical and data processing skills from researchers, many studies that utilize GPS measurements are generally small in size. Furthermore, depending on the technology used, some activities, such as swimming or cycling in natural environments, may be difficult to capture. Generalizability by socioeconomic status must also be taken into account if participants are required to have a smartphone to participate. Many privacy issues must be considered when gathering GPS data, as, in some countries, these data represent protected health information because they are easily identifiable. Additionally, GPS data can only detail time spent in nature and cannot quantify an individual’s experience in that location and cannot independently identify activities while in nature. For example, GPS data may show someone that walked through a park, but it cannot reveal how they were interacting with nature, unless the study design includes a component that queries individuals about time spent and experience with walking through the park [49].

### 3.2. Selected Population-Level Estimates of Time Spent in Nature

Population-level data on time spent outdoors (a commonly used metric collected by self-report) is one indicator of global nature exposure and is useful in examining associations between time in nature and health on a large scale. As outlined above, most studies record time spent outdoors versus time spent in nature. Therefore, we present results on current estimates of both time spent outdoors and time spent in nature. Through a literature review, we identified ten national surveys (Table 1) of populations within the United States (3), Canada (2), the United Kingdom (3), Australia (1) and New Zealand (1), which examined time spent outdoors or time spent outdoors in nature [31,32,33,34,35,42,43,50,51,52]. Sample sizes ranged from 594 participants in the Canadian Health Measures Survey to 468,000 individuals in MENE in England. Study populations were commonly representative of the general population; however, there were instances in which questionnaires were restricted to specific subgroups, such as children aged three to six years (via parent report). We also identified a cross-sectional, multinational survey (BlueHealth International Survey [BIS]) of 18 countries with a sample size of 18,838 individuals that included information about visits to and time spent in green and blue spaces [53]. Though estimates of time spent in natural environments from the BIS are not yet publicly available, it is important to highlight that a multinational effort to measure time spent in natural environments does exist.

Specifications of self-report varied across surveys. In four of the 10 questionnaires [31,42,43,52], participants were asked to report time spent specifically in natural environments, for example, by using their own definition of nature or provided definitions of outdoors, such as open spaces in and around towns and cities, including parks, canals and nature areas, beaches, and the countryside, including farmland, woodland, hills and rivers. The other six surveyed time spent outdoors more broadly, which is not necessarily equivalent to time spent in nature or a natural environment [32,33,34,35,50,51].

Across all the studies, exposure to nature and/or the outdoors was measured as hours in a typical week spent in nature, hours spent outdoors in the past week, average hours per week spent doing outdoor recreation activity, occasions in the last week spent outdoors, and 24-h recall diaries used to estimate time-activity patterns. Reports most commonly quantified duration of exposure to nature, and three surveys (MENE, National Survey for Wales, and The National Human Activity Pattern Survey) included questions regarding frequency [35,42,52]. Only Scotland’s People and Nature Survey gathered information on intensity via perception statements, such as “My local green space is somewhere I can relax and unwind,” “My local green space is an attractive place,” and “My local green space allows me to explore nature on my doorstep” [43].

In the United States, the National Human Activity Pattern Survey found that on average 87% of daily time was spent indoors [35]. The National Kids Survey reported that most children (62.5%) were spending at least two hours of time outdoors daily [32]. Only one US-based study asked participants to report time spent specifically in nature [31]. During a typical week, about half of adults surveyed reported spending between zero and five hours outside in nature, and the majority reported spending fewer than 10 h outside in nature per week.

Similarly, in Canadian study populations, researchers reported that, on average, participants spent the majority of time indoors (88.9%), with limited time outdoors (5.8%) [50]. In a survey of approximately 600 children aged three to six years included in the Canadian Health Measures Survey, on average, children spent approximately two hours per day outside and about half of this time occurred during daycare/school hours [57]. In UK populations, nearly two thirds of adults visited the natural environment at least once a week [58]. Results from the MENE indicated that, on average, nature visits took place within 4.9 miles of the home, 86% of visits were less than three hours in duration, and 52% of visits were taken to green space in a town or city. Similarly, in Wales, participants reported spending up to an hour (28%), one to two hours (36%), or two to three hours (18%) on average per visit; outdoor visits were most frequent to local parks or other local space (25%), woodlands or forests (17%), and beaches or coastlines (19%) [52].

While national surveys can provide a large amount of information, it is important to note that these do have some limitations. These surveys are observational, and results could be biased (e.g., selection bias, recall bias). For example, survey respondents may have been more likely to participate due to previously having a fondness of nature (i.e., selection bias). Similar to questionnaires, national surveys may not adequately capture how one interacts with nature. Studies need to assess the duration of exposure, the seasonality of exposure (e.g., the role of deciduous trees being in leaf, physical comfort level in relation to the season), and the quality of nature (e.g., mature trees versus immature trees versus shrubs versus lawn) [5].

The studies reviewed above present a rough baseline of how much time children and adults are spending in nature across the examined countries; however, more information is required to have an accurate estimate. Furthermore, these surveys may not be representative, as they do not capture the entire population of each country. As previously noted, many surveys do not explicitly ask about time spent in nature and instead opt to ask about time spent outdoors. Finally, the studies that do examine time spent in nature were not designed to ascertain the amount of time spent in nature, but rather the effects that nature may have on those that do spend time in nature. It is important to note that other studies or surveys of time spent in nature or outdoors may exist in other countries, though these were outside the scope of this review.

## 4. Data Gaps and Limitations

There are several limitations on how researchers have previously measured time spent in nature. First, nature is not one specific construct, and defining nature across studies is complex and inconsistent. When researchers talk about “time spent in nature,” there is a need for more specificity. Researchers must carefully consider how to best define and parameterize nature exposure and time spent in nature based on the research question and include quantitative and qualitative components where applicable. Nature and health may be linked through multiple mechanisms; thus, it is critical to elucidate when, where, and for whom these mechanistic pathways operate in order to determine how best to design and manage spaces to maximize health. As an example, future work should examine nature–health relationships by age, since children and elderly populations may perceive and react to nature in different ways.

In addition, research should consider nature at different scales, since small natural elements at the neighborhood level, such as street landscaping, are often neglected [6]. Measures such as greenness around the residential address and access to green space from the home are imperfect proxies for time spent in those locations and may not accurately capture how much time individuals spend in nature [59]. Questionnaires and surveys are other commonly used measures, but that may suffer from recall and/or social desirability bias. Individuals may have trouble accurately recalling how much time they spend in nature or may respond with what they believe researchers want to hear. Cultural and geographical factors on how one defines nature may affect responses across different cultures and geographies, leading to issues with comparability [59]. Furthermore, it is critical to assess how and why time spent in nature varies by factors such as race/ethnicity, socioeconomic status, and urbanicity.

While GPS tracking may be objective, it also suffers from limitations regarding how researchers define nature and the generalizability of people willing to participate in such studies. In addition, cost and data processing requirements are higher with GPS data, which limits the availability of this approach [60].

Another major limitation is that existing measures are almost all cross-sectional, rather than longitudinal, resulting in poor data on trends in nature contact and limited ability to assess the impacts of population or individual-level interventions. Existing data on nature contact and health generally lies on a spectrum ranging from great exposure–poor outcome data to poor exposure–great outcome data. For instance, the Scottish Nature Survey gathers rich data on time spent in or interaction with nature but poor health outcome data, whereas the Scottish Health Survey collects poor information on exposure (i.e., a single question on green exercise) and great outcome data (e.g., biomarkers). The gap between these two situations is a key limitation in existing data and should drive the design and implementation of future research in this area.

While the focus of this review was on visual exposure to nature, it is critical to note that humans experience nature contact through multiple senses (e.g., sound, smell, taste, touch) [61]. Though research on these sensory pathways is emerging, there are many shortcomings in understanding that can be further explored. For instance, the visual pathway has been well researched [61], though less is known about the impacts of nature contact for individuals who are visually impaired [62]. Furthermore, recent research indicates that exposure to “natural soundscapes” (e.g., animal sounds, water sounds) is associated with improved health outcomes, positive affect, and decreased stress [63]; however, the majority of these studies occur in laboratory settings and are limited by the extent to which they assess the impacts of diversity of soundscapes on health outcomes.

## 5. Future Directions

Research on the exposure assessment of nature contact, including the methodologies described in this text used to quantify (via metrics of duration or frequency in nature) and qualify (e.g., surveys evaluating nature experience or connection to nature) human interaction with nature are important in predicting health benefits. Collaboration across national and cross-national entities to administer national and global health surveys (e.g., existing surveys including the Behavioral Risk Factor Surveillance System (BRFSS), National Health Interview Survey (NHIS), and National Health and Nutrition Examination Survey (NHANES) in the United States or the Health Survey for England (HSE) combined with representative data on duration and frequency of nature exposure with intensity data on nature from satellite or land use spatial datasets would provide rich information. The International Physical Activity and the Environment Network (IPEN) Adult Study, which assesses the relationships between perceived neighborhood built environmental attributes (including greenness and park access) and health outcomes across twelve countries [64], provides an opportunity for improving data collection and linkage at the population level across different geographies. Some countries have centralized health records that could be used to link individual health information with individual survey responses. In the UK, for example, where everyone has a National Health Service record, there is huge potential to link individual survey responses to those health records in a secure setting. Moving forward, creating harmonized constructs of how we define nature across studies and different global regions would enable easier comparisons between countries.

With the growing ubiquity of location-enabled smartphones, researchers could capitalize on novel data streams to reveal further insights into nature contact. Mobile applications could gather GPS data to create participant-specific estimates of time spent in nature and could also incorporate measures such as experience sampling methods (ESM) and ecological momentary assessments (EMA) that send participants short surveys in real-time that ask whether and how participants are interacting with their surroundings or ascertain participants’ moods while they are in nature [65,66,67]. ESM/EMA is emerging as a promising tool that could also be used to more accurately quantify exposure to greenness, as well as accurately categorize the type of greenness exposure (e.g., field versus forest versus public park) by asking participants to describe their surroundings [49,68].

In addition, combining concurrent measures of accelerometry and other participant wearables might provide more accurate information on levels of physical activity in natural environments. Finally, the majority of research on time spent in nature is conducted in Western and higher-income countries. To effectively capture the diversity of exposures, experiences, and populations that might benefit from nature exposure, the field must broaden to cover more countries and more diverse study populations.

Fine-tuning metrics of nature exposure such as those described in this review and crafting novel methods to measure the complexities of nature contact will allow increased accuracy and larger applicability for investigating health benefits of nature. Improved exposure assessment in this field can lead to interventions in diverse populations with varying accessibilities, quality, and relatedness to greenness. Innovative policies, including park prescriptions (i.e., ParkRx or NatureRx programs where physicians prescribe that their patients spend more time in nature) or greening schoolyards or vacant lots, offer a framework within which the metrics of nature contact can be used in conjunction with health outcomes to improve health and provide an optimal dose of nature [69,70,71,72,73].

## 6. Conclusions

The purpose of this review was to examine the literature on exposure assessment methods for measuring time spent in nature, discuss current population-level estimates of time spent in nature, and identify the strengths, limitations, and future research directions in this area. Many researchers employ proxies for measuring time spent in nature, with the most common methods including surrounding greenness, self-reported questionnaires, and GPS tracking, each of which has its limitations. Some population-level surveys of time spent outdoors or time spent in nature exist, though these are limited by cross-sectional design, susceptibility to selection and recall bias, and often poor quality or sparse measures of health with which to assess impacts. Expanding exposure assessment to include multiple components (e.g., time spent in nature, frequency of visits to nature, experience with nature) of current methods will provide a more comprehensive understanding of the health effects of nature exposure and will inform health policy and urban planning.

## Figures and Tables

**Table 1:** Population-level data on time spent outdoors in nature collected in ten national surveys of populations within the United States, Canada, the United Kingdom, Australia and New Zealand.

National Survey	Year	Geography	Population	Sample Size	Definition of ‘Nature’	Measure of Exposure	Major Results
The Nature of Americans: National Report	2012	US	American adults, children, and parents	11,817 individuals	Instructed to use own definition of nature	Hours in a typical week spent outside in nature (not including organized sports)	Time spent in outdoor activities averaged 6.6 h in a typical week and declined slightly with age from 6.7 h among eight-year-olds to 5.6 h among 12-year-olds.
Children’s Time Outdoors: Results and Implications of the National Kids Survey	2007–2009	US	General population	3000 households (1450 youth)	Outdoor time	Hours in the week preceding household interview spent outdoors	Most children spent at least two hours outdoors daily during the week preceding the household interview (62.5 % of children spent two or more hours outdoors on a weekday, 78.2% on a weekend).
Missing Trees: The Inside Story of an Outdoor Nation	2013	AUS	Australians aged 14 to 64	1002 individuals	Outdoor time	Average hours per week spent doing outdoor recreation activities	One in three respondents aged 14 to 64 years spent on average less than two hours per week doing outdoor recreational activities, such as gardening, playing sport outdoors, taking the kids to the park or walking the dog.
Canadian Health Measures Survey	2012–2013	CA	Children aged three to six years (parent report)	594 individuals	Outdoor time	Parental report of outdoor time	On average, participants spent approximately 2 h/day outside and about half of this time occurred during daycare/school hours.
Canadian Human Activity Pattern Survey 2 (CHAPS 2)	2010–2011	CA	General population	5011 individuals	Outdoor time	24-h recall diary information used to estimate time-activity patterns	A majority of the time was spent indoors (88.9%), most of which was indoors at home, with limited time spent outdoors (5.8%).
Monitor of Engagement with the Natural Environment: The national survey on people and the natural environment (MENE)	2009–2018	UK (England)	General population	~46,000 individuals per year (468,000 total)	Outdoor time (time in open spaces in and around towns and cities, including parks, canals and nature areas; the coast and beaches; and the countryside including farmland, woodland, hills and rivers, does not include time in your own garden)	Occasions in the last week spent outdoors	During the 12 months from March 2017 to February 2018, nearly two thirds of adults living in England visited the natural environment at least once a week (62%). A significant proportion took visits less than once a month or never took visits (18%). On average, nature visits took place within 4.9 miles of the home, 86% of visits were less than 3 h in duration, and 52% of visits were taken to greenspace in a town or city.
How New Zealanders distribute their daily time between home indoors, home outdoors and out of home	2015	NZ	General population	445 households (1026 individual participants)	Outdoor time	Time spent in microenvironments in one day	Individuals spent on average 15.85 h per day at home indoors and 0.54 h per day at home outdoors.
The National Human Activity Pattern Survey (NHAPS)	1992–1994	US	General population	9386 individuals	Outdoor time	24-h recall diary containing beginning and ending times, activity, location, presence of a smoker, time spent	Approximately 87% of time was spent indoors, 5 to 6% spent in a vehicle, with the remaining 7 to 8% spent outdoors.
Scotland’s People and Nature Survey (SPANS)	2013/2014, 2017/2018, 2019/2020,	UK (Scotland)	General population	10–12,000 individuals per wave	‘Outdoors’ includes mountains, moorland, farmland, forests, woods, rivers, lochs and reservoirs, beaches and the coast and open spaces in towns and cities. ‘Visits to the outdoors’ refers to leisure trips taken from home or while away from home on holiday, provided the holiday was in Scotland.	In the last 12 months, outdoor recreation frequency and volume (monthly); outdoor recreation location and activity (bi-monthly), travel distance, duration, use (quarterly); motivations and benefits associated with visiting the outdoors (bi-annually); access issues (annually).	Between May 2019 and March 2020 [54], the proportion of adults visiting the outdoors on a regular weekly basis also increased, up to 63% from 57% in 2017/2018 and 50% in 2013/2014. Over a quarter of respondents in 2019/2020 reported that they had visited the outdoors on a daily basis (26%). The most frequently cited reasons for visiting the outdoors were exercising a dog (42% of visits) and health and exercise (41%). Around a third of visits were taken to relax or unwind (34%), while just over a fifth of visits (22%) were taken to enjoy fresh air or pleasant weather. Nearly half of all outdoor visits were taken to green spaces within towns and cities (49%), two fifths in the countryside (41%) and 10% in seaside locations. Three-quarters of adults strongly agreed that their most recent outdoor visit helped them to relax and unwind (75%), and nearly as many strongly agreed that the experience improved their physical health (71%) or that the visit had made them feel energized/revitalized (69%).
National Survey for Wales—Outdoor Recreation	2016, 2018, 2020	UK (Wales)	General population	10,000 individuals per wave	Outdoor visits and outdoor recreation	In the last 12 months or four weeks, the number, length of time spent, use/activity, location, travel distance, and motivation for outdoor visits; access to a garden; outdoor recreation number of visits, use/activity, and length of time spent.	In the 2018 wave, approximately 81% of adults took part in one or more outdoor visits or recreation activities in the past 12 months, with a mean of 13.1 visits over the last four weeks. Outdoor visits were most frequent to local parks or other local space (25%), woodlands/forests (17%), and beach/sea/coastline (19%). The duration of outdoor visits varied, with most participants indicating spending up to an hour (28%), between one to two hours (36%), or two to three hours (18%) on average per visit.
BlueHealth International Survey (BIS)	2017–2018	Multinational (United Kingdom, Ireland, France, Spain, Portugal, Germany, Netherlands, Czech Republic, Italy, Sweden, Finland, Estonia, Greece, and Bulgaria, Hong Kong, Canada, Australia (Queensland), USA (California)	General population	18,838 individuals (~1000 per country)	Green and blue space visits	In last four weeks, number of green/blue space visits; date of most recent green/blue space visit, type of green/blue space visited, time of day of visit, time spent in green/blue space, perceived quality of green/blue space, motivation for visit, main activity/activity, duration, travel distance to green/blue space.	Specific estimates of time spent in green and blue spaces are forthcoming, though some research on green/blue space exposure and health outcomes using this survey data is available [55,56].

## Data Availability

Not applicable.
